# Immunotherapies: Exploiting the Immune System for Cancer Treatment

**DOI:** 10.1155/2018/9585614

**Published:** 2018-03-14

**Authors:** Jeffrey Koury, Mariana Lucero, Caleb Cato, Lawrence Chang, Joseph Geiger, Denise Henry, Jennifer Hernandez, Fion Hung, Preet Kaur, Garrett Teskey, Andrew Tran

**Affiliations:** Graduate College of Biomedical Sciences, Western University of Health Sciences, Pomona, CA 91766, USA

## Abstract

Cancer is a condition that has plagued humanity for thousands of years, with the first depictions dating back to ancient Egyptian times. However, not until recent decades have biological therapeutics been developed and refined enough to safely and effectively combat cancer. Three unique immunotherapies have gained traction in recent decades: adoptive T cell transfer, checkpoint inhibitors, and bivalent antibodies. Each has led to clinically approved therapies, as well as to therapies in preclinical and ongoing clinical trials. In this review, we outline the method by which these 3 immunotherapies function as well as any major immunotherapeutic drugs developed for treating a variety of cancers.

## 1. Introduction

As long as the fight against cancer remains an uphill battle, there will be an adamant drive for the development of aggressive therapeutics aimed at minimizing or inhibiting cancer cell proliferation and metastasis. The efficacies of traditional treatment modalities for the management of cancer, such as radiation therapy and chemotherapy, are often limited by the occurrence of severe toxicities, which account for the numerous side effects experienced by oncology patients [1]. Radiation therapy is an effective means for systemic treatment; however, localized collateral damage of healthy tissues occurs as a consequence. Chemotherapeutic agents, such as genotoxic drugs or antimetabolites, reveal short-term side effects and are often administered in combination with surgical interventions [2]. Although surgical excision of tumors is effective only in early stages of disease, it loses its effectiveness once the malignancy becomes metastatic.

Cancer immunotherapy has become a staple of modern oncology since the first immunotherapy was described in 1985. Immunotherapeutic approaches utilize components of a patient's own immune system to selectively target cancer cells thereby mitigating many of the side effects associated with traditional treatment options. The immune system can detect cancer cells in one of two ways: by recognizing molecules uniquely expressed in cancer cells (tumor-specific antigens or mutations) or by recognizing molecules that are differentially expressed in cancer cells relative to normal cells (tumor-associated antigens) [3]. Immunotherapy is an effective and promising treatment option for cancer due to its selectivity and long-lasting effects and demonstrated improved overall survival and tolerance [4].

High-dose interleukin 2 (HD IL-2) was the first reported immunotherapy capable of mediating a long-term and complete response (CR) in patients with advanced melanoma and renal cancer [5, 6]. Phase II clinical trials demonstrated that 9 patients (7%) with metastatic melanoma and 10 patients (7%) with metastatic renal cell cancer treated with biologic therapy of HD IL-2 achieved complete regression of disease with hypotension, secondary to underlying capillary leak, being the most commonly reported toxicity [7–9]. These early studies substantiated that altering host immune responses with exogenous immune effectors could safely mediate antitumor effects on a subset of patients with advanced malignancies [7, 8, 10]. FDA approval of HD IL-2 for the treatment of patients with renal cancer and melanoma was granted in 1992 and 1998, respectively [7, 8, 10], which established immunotherapy as the newest paradigm for the treatment of cancer.

In the decades following FDA approval of HD IL-2, there have been unprecedented advancements regarding the cellular and molecular drivers of tumorigenesis and the mechanisms through which tumorigenic cells circumvent destruction by the immune system [8]. More recently, three distinct therapeutic modalities have revolutionized the field of immunooncology: checkpoint inhibitors, adoptive T cell transfer, and bivalent antibodies.

## 2. Checkpoint Inhibitors

Cancer cells have adapted specialized cellular mechanisms to facilitate the development of the tumor microenvironment [11]. One method tumor cells employ to ensure their survival and progression is to evade immune system checkpoints [12]. Immune system checkpoints function to monitor autoimmunity and mitigate collateral tissue damage due to immune responses by modulating costimulatory and inhibitory signaling [13]. However, during tumorigenesis, the dysregulation of checkpoint protein expression can result in the aberrant activation of inhibitory checkpoint receptors thereby preventing T cells from recognizing and eliminating tumorigenic cells [12–14].

Checkpoint inhibitors are a class of immunotherapies that induce a T cell-mediated antitumor responses by selectively blocking the inhibitory checkpoint receptors subject to manipulation by cancer cells [15]. The immune checkpoint receptors that have served as the primary targets of clinical cancer immunotherapy include the following: cytotoxic T lymphocyte-associated antigen 4 (CTLA-4), programmed cell death protein 1 (PD-1), programmed cell death 1 ligand 1 (PD-L1), lymphocyte activation gene 3 (LAG-3), B and T lymphocyte attenuator (BTLA), and T cell immunoglobulin and mucin protein 3 (TIM-3) [13, 16].

### 2.1. Anti-CTLA-4 Treatment

The first immune checkpoint receptor to be clinically targeted was cytotoxic T lymphocyte-associated antigen 4 (CTLA-4) [17]. CTLA-4 is an inhibitory immune checkpoint receptor expressed on the surface of activated T cells and regulatory T cells that binds to B7 family ligands (CD80 and CD86) on antigen-presenting cells [17, 18]. CTLA-4 functions to downregulate T cell proliferation by outcompeting CD28, a costimulatory receptor, for ligand binding and recruitment of serine/threonine phosphatase [19]. Anti-CTLA-4 relieves the natural brakes on T cells, thus allowing them to perform their effector function for an extended period of time [20].

Anti-CTLA-4 antibodies potentiate an antitumor response by blocking inhibitory CTLA-4 receptors to facilitate T cell activation [21, 22]. Ipilimumab, a monoclonal anti-CTLA-4 antibody, was the first checkpoint inhibitor to demonstrate an improved overall survival rate in patients with previously treated metastatic melanoma [21, 23]. In 2010, Hodi et al. reported that patients with metastatic melanoma treated with ipilimumab in combination with a gp100 peptide vaccine experienced an increased objective response rate (35 patients or 6.5%) relative to the control group (2 patients or 1.5%). Additionally, patients who were administered ipilimumab with or without the gp100 peptide vaccine experienced an increased median overall survival from 6.4 months to 10 months relative to the control group [21]. The most common immune-related toxicity associated with ipilimumab administration at any grade was diarrhea, which was reported by 31% of patients. Immune-related severe adverse events (SAE) were reported in 10–15% patients: skin rash, diarrhea and colitis, hepatitis, and endocrinopathies. There were 7 deaths due to immune-related toxicities; however, the majority of the SAE were readily reversible with appropriate treatment [21]. Based on the findings of this clinical trial, ipilimumab was awarded FDA approval thus substantiating the validity of checkpoint inhibitors as a therapeutic option for the treatment of metastatic melanoma. Furthermore, the durability of ipilimumab responses was verified by a longitudinal follow-up study of 177 patients treated with ipilimumab in three separate clinical trials; this study reported potentially curative tumor regression in a small percent of patients with metastatic melanoma [24].

### 2.2. Anti-PD-1/PD-L1 Therapies

**Figure 1:**
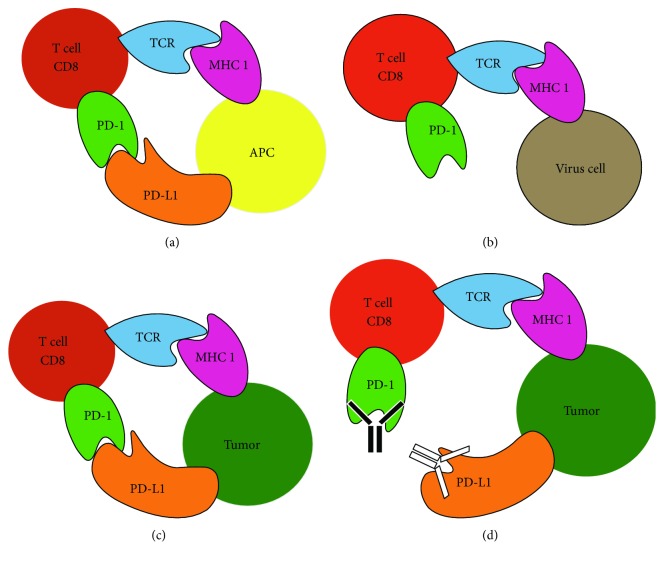
PD-1/PD-L1's function in normal and tumor environments. (a) PD-1 on T cells binds to a PD-L1 ligand on APC, deactivating the T cell. (b) PD-1 is upregulated in exhausted T cells. (c) Tumor cells express PD-L1, as a survival tactic, which engages with PD-1 expressed by tumor antigen-specific T cells, and deactivate the T cell. (d) Checkpoint inhibitors targeting PD-1 and PD-L1 prevent the tumor cell from binding to PD-1 and thus allow T cells to remain active.

Under physiological conditions, PD-1 interacts with PD-L1 present on activated CD8^+^ T cells to inhibit further antigen-mediated T cell activation [25]. Many tumor cells express PD-L1, while many tumor antigen-specific T cells express the complementary PD-1 receptor (Figure 1) [26]. Checkpoint inhibitors targeting PD-1 and PD-L1 have demonstrated positive clinical effects on more than 15 cancer types [27]. Due to their success, the FDA has approved two anti-PD-1 monoclonal antibody therapies, nivolumab (Opdivo) and pembrolizumab (Keytruda), for the treatment of specific cancers [26].

Pembrolizumab, a humanized anti-PD-1 antibody developed by Merck, was the first to be awarded US FDA approval in September 2014 and has since then been approved for the treatment of metastatic NSCLC [28]. Treatment of NSCLC with pembrolizumab is contingent upon PD-L1 expression of the tumor. In a 495-patient clinical trial, PD-L1 expression in at least 50% of the tumor cells correlated with a marked improvement in pembrolizumab efficacy.

In 2012, a 296-patient clinical trial described an objective response in patients with NSCLC, melanoma, and renal cell cancer when treated with anti-PD-1 nivolumab, an IgG4 human monoclonal antibody [29]. Nivolumab later gained US FDA approval in December 2014 for the treatment of unresectable metastatic melanoma based on a phase 3 randomized, controlled open-label study called Checkmate-037. In the study, 370 patients were enrolled with 268 receiving 3 mg/kg nivolumab and the remainder receiving chemotherapy treatment (*N* = 102). The trial identified a 32% overall response rate for the 3 mg/kg treatment dose in patients with unresectable or metastatic melanoma who had previously received ipilimumab therapy or if relevant a BRAF inhibitor [30]. Following its breakthrough approval in 2014, nivolumab has since received FDA approval for the treatment of metastatic squamous NSCLC that had progressed with platinum-based chemotherapy [30].

In a study involving nivolumab administration to treat soft tissue and bone sarcomas, 28 patients with metastatic or unresectable sarcomas were treated with nivolumab in conjunction with or without the tyrosine kinase inhibitor pazopanib [31]. Of the 24 eligible patients, half exhibited a partial response or a stabilization of the disease after at least 4 cycles of nivolumab injection [31]. While the study was small and retrospective, the results confirm the use of checkpoint inhibitors for treating soft tissue and bone sarcomas [31].

Beyond its use in the treatment of a multitude of generic malignancies, PD-1 blockade has been shown to be particularly successful in targeting tumors containing high tumor mutation burdens. Tumor mutation burden (TMB), the total number of mutations per area of the genome, has been identified as a potentially important and sensitive biomarker in predicting therapeutic responses with multiple classes of treatment for cancer [32, 33]. Mismatch repair-deficient tumors, which contain mutations in genes responsible for correcting the single-base pair mismatches and insertion-deletion loops during replication, have been shown to have a high TMB and strong response to PD-1 blockade [33]. In initial studies of colorectal cancer, only 1 case in 33 responded to treatment with PD-1 blockade, whereas a targeted study of colorectal cancer that had mismatch repair-deficient tumors, and therefore high TMB, demonstrated a strong response. In this case, progression-free survival rate at 20 weeks was 78% versus 11% in the mismatch repair-proficient group [33]. Tumors with a high TMB are more likely to express neoantigens that can attract effector T cells, making them more viable candidates for PD-1 blockade treatment [34].

The presence of the PD-1 ligands (PD-L1 and PD-L2) on the membrane of tumor cells has been shown to be an important and obvious, but not definitive, biomarker for predicting a tumor response to PD-1 blockade [33, 35]. PD-1 is upregulated after T cell activation, but with chronic illnesses, such as cancer, long-term upregulation of PD-1 and a high rate of interaction with PD-1 ligands can cause a decrease in effector capacity and proliferation. This phenomenon in T cells is known as “T cell exhaustion.” In some cancers, such as melanoma, it is believed that T cell recognition of the tumor results in the expression of INF-*γ* by T cells, which leads to the expression of PD-L1 by tumor cells, which in turn inhibits the T cell antitumor response [36]. It is for this reason that melanoma was selected early for PD-1 blockade treatment, leading to the approval of the aforementioned pembrolizumab and nivolumab by the FDA in 2014 [37]. This is mainly marked by NSCLC and melanomas with many other tumors being immune-inhibitory without expressing PD-L1.

One biomarker that has been identified in a subset of PD-1 blockade responsive cancers, which oddly have normal expression of PD-L1/PD-L2, is microsatellite instability (MSI) [36, 38]. MSI is diagnosed by the variable length of microsatellites, which is likely the result of epigenetic silencing or mismatch repair deficiency [36]. It is associated with high TMB and has been shown to occur in colorectal (20%), endometrial (22–33%), cervical (8%), and esophageal (7%) cancers [38]. These tumors persist despite the high number of neoantigens that result from MSI and low tumor cell expression of PD-L1, with one hypothesis being that there is a correlation between MSI and PD-L1^+^ myeloid cell concentrations. It is thought that the high rate of interaction between PD-1 on T cells and its ligand on the myeloid cells results in T cell exhaustion [36].

In a study using next-generation sequencing (NGS) of archived melanoma tissues treated with PD-1 blockers, it was shown that patients who responded to the treatment had a higher TMB [39]. Specific mutations have now been correlated with high TMB, including NF1 mutations in melanoma [39] and PMS2 mutations in melanoma and squamous cell carcinoma [40]. Basal cell carcinoma and cutaneous squamous cell carcinoma often have a high TMB, as a result of UV light exposure, a major risk factor [41, 42]. In a recent phase I trial, one patient with metastatic basal cell carcinoma and one patient with metastatic squamous cell carcinoma were treated with REGN2810, a fully human anti-PD-1 monoclonal antibody [43]. The patient with metastatic basal cell carcinoma experienced a partial response that persisted through at least 12 months, whereas the patient with metastatic cutaneous squamous cell carcinoma experienced a complete response and showed no clinical or radiographical evidence of disease after 16 months [43]. The study concluded that a high TMB can elicit antitumor cellular immunity unleashed by PD-1 blockade. Interestingly, the patient who experienced a partial response had previously been treated with a hedgehog inhibitor, which has been associated with an influx of cytotoxic T cells and could potentiate antitumor immune responses. Future research may further examine the synergistic effects between hedgehog inhibitor therapy and anti-PD-1 blockade [43].

The study notes that a reductionist's view would anticipate that other UV-associated tumors with high TMB would be more responsive to PD-1 blockade, though there are numerous other variables to consider. It should be noted that this association of high tumor mutational load with PD-1 response is substantiated mainly in lung cancer and melanoma, with cases in other malignancies showing opposing results.

### 2.3. Anti-CTLA-4/Anti-PD-1 Combinatorial Therapy

Although effective as standalone treatments, the mechanism of action for anti-CTLA-4 and anti-PD-1 antibodies is distinct and nonoverlapping. Both CTLA-4 and PD-1 negatively regulate T cell activation, but CTLA-4 does so by mediating the inhibition of Akt phosphorylation via PP2A while PD-1 inhibits Akt phosphorylation by preventing CD28-mediated activation of PI3K [44]. Thus, clinical paucities in anti-PD-1 treatment can be addressed with a reliable anti-CTLA-4 treatment and vice versa. For example, despite CTLA-4 blockade, PD-1/PD-L1 interaction can perturb T cell proliferation and cytokine production. For this reason, nivolumab and ipilimumab are often used to complement each other, particularly in treating advanced melanoma [45, 46]. This combination therapy achieved a greater progression-free survival rate (11.5 months) as compared with ipilimumab monotherapy (2.9 months) and nivolumab monotherapy (6.9 months) [45].

The most apparent drawback with this combinatorial treatment is the commensurate increase in notable adverse effects. In the same clinical trial, 27% of patients in the ipilimumab monotherapy group, 16.3% in the nivolumab monotherapy group, and 55% in the combination therapy group exhibited grade 3/4 adverse effects. Interestingly, sequential inhibition of CTLA-4 followed by anti-PD-1 treatment does not seem to provoke immune-related adverse effects [47]. Despite this fact, anti-PD-1/anti-CTLA-4 combination therapy is being used a first-line treatment for previously untreated patients with metastatic melanoma.

### 2.4. Novel Checkpoint Inhibitors

Despite the successes of anti-CTLA-4 and ant-PD-1 therapies, these therapeutic modalities are capable of producing a durable response in a small subset of cancer patients. However, during the last decade, several additional checkpoint receptors have been identified for their potential to serve as novel targets for cancer immunotherapeutics. The new generation of checkpoint inhibitors which have generated promising clinical and preclinical results includes lymphocyte activation gene-3 (LAG-3), B and T lymphocyte attenuator (BTLA), and T cell immunoglobulin and immunoreceptor tyrosine-based inhibitory motif domain (TIM-3).

LAG-3 or CD223 is a coinhibitory receptor expressed on various lymphoid cells including activated T cells and regulatory T (T-Regs); LAG-3 inhibits effector T cell killing by inducing T-Reg-mediated immune suppression [48–50]. Concurrent blockade of LAG-3 and PD-1 has been shown to restore the immune function of exhausted CD8^+^ T cells to augment a potent antitumor response, while exhibiting an improved safety profile as demonstrated by a significant decrease in the occurrence of systemic toxicities relative to anti-CTLA-4 treatments [50]. Additionally, multiple clinical trials have demonstrated the validity of LAG-3 as a vaccine adjuvant for melanoma and prostate cancer as well as of LAG-3 in combination with chemotherapy for the treatment of metastatic breast cancer [51–53].

BTLA is an additional coinhibitory receptor expressed on lymphoid cells that has produced propitious preclinical results. In melanoma, BTLA participates in cross-activation (cross-talk) with herpesvirus entry mediator (HVEM), a tumor necrosis factor receptor, to induce a BTLA-dependent T cell inhibition [54]. Preclinical results demonstrate that monoclonal anti-BTLA antibodies can promote T cell activation in melanoma patients by preventing BTLA/HVEM coinhibitory signaling, although safety profiles have yet to be established [55, 56].

TIM-3 is a membrane receptor expressed on T helper 1 (Th_1_) cells that binds to galectin-9, a ligand upregulated in breast cancers and melanomas [57, 58]. In tumorigenic cells, TIM-3/galectin-9 signaling inhibits Th_1_ cell immune responses by inducing T cell exhaustion [58, 59]. Similar to LAG-3, preclinical trials have shown that concurrent blockade of TIM-3 and PD-1 reverses TIM-3/galectin-9-induced T cell exhaustion to potentiate an antitumor response and reduce tumor burden [58].

This new generation of immune checkpoint inhibitors has shown promising results that could potentially broaden the use of biologic therapeutics for the treatment of various forms of cancer and address the deficiencies that exist in cancer immunotherapy.

## 3. Adoptive T Cell Transfer

The adoptive cell transfer (ACT) technology takes advantage on the reliance of immune cells in surrounding the tumor environment, stimulating cells ex vivo, and manipulating the immune environment for the introduction of effector cells [60–62]. ACT typically consists of three parts: lympho-depletion, cell administration, and therapy with high doses of IL-2. Lympho-depletion using chemotherapy or radiation has proven to enhance the antitumor effects of transferred lymphocytes [63]. It was also shown that IL-2 was crucial for the expansion of the transferred lymphocytes ex vivo, as well as for the regression in metastatic melanoma when directly administered [61, 64].

### 3.1. Tumor-Infiltrating Lymphocytes (TILs)

**Figure 2:**
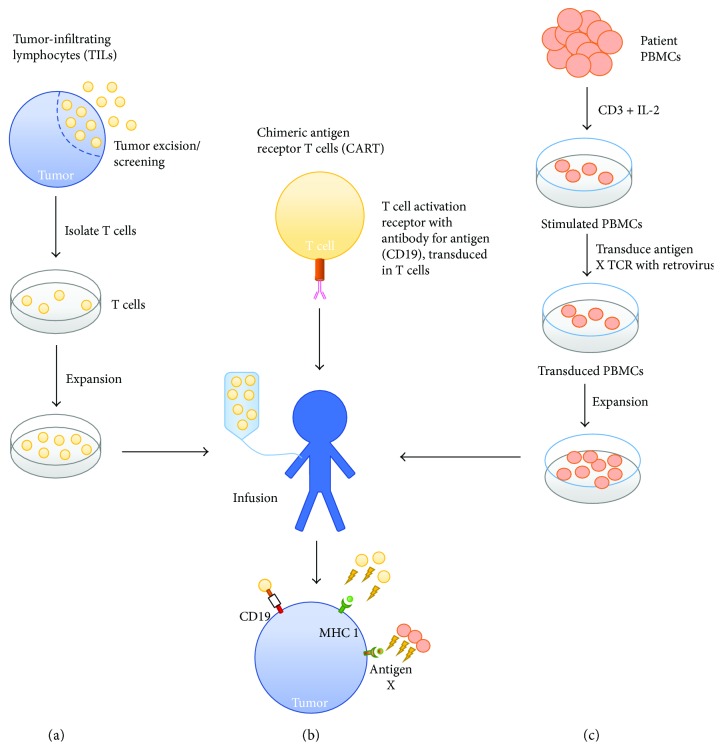
Procedure for adoptive T cell transfer with CAR T cells and TILs. Adoptive transfer of TILs and CAR T cells to target the MHC1 and CD19 antigen on a tumor cell, respectively. (a) Excision of the tumor followed by isolation and expansion of lymphocytes present from the excised tumor. Expanded cells are then infused into the patient. (b) Chimeric antigen receptor T cells are designed to target the CD19 antigen and are then infused into the patient. (c) TCR-transduced T cells are generated from patients' PBMC and stimulated with a retrovirus containing the antigen “X”-specific TCR.

One form of transferred lymphocytes is tumor-infiltrating lymphocytes (TILs) which were discovered to be mononuclear lymphocytes that had a propensity to surround and invade tumors [65]. These TILs were first discovered in resected melanomas and were found to contain a mixture of both CD4 and CD8 T cells. The general procedure for autologous TIL therapy is stated as follows: (1) the resected melanoma is digested into fragments; (2) each fragment is grown in IL-2 and the lymphocytes proliferate destroying the tumor; (3) after a pure population of lymphocytes exists, these lymphocytes are expanded; and (4) after expansion up to 10^11^ cells, lymphocytes are infused into the patient (Figure 2(a)) [66]. Adoptive T cell transfer of TILs produces a 50% cancer response rate and 20% complete response rate in metastatic melanoma, and since the responses are very durable, the 20% complete response rate translates into a 20% cure rate. Before the recent development of checkpoint modulators (anti-PD-1), which shows a comparable level of response, TILs had been the only agent approved by the US FDA for patients with metastatic melanoma [67–71].

In a similar vein, T cell transfer through T cell receptor modifications has shown promise, especially with targeting common tumorigenic mutations, such as Ras mutations. The significance of Ras mutations in cancer has been well identified and acknowledged for several years. Consequently, Ras is considered an alluring target for cancer therapy as it is commonly mutated in cancer development. In addition, the mutation usually occurs at the onset of tumorigenesis, thus resulting in high expression throughout nearly all tumor cells. Mutations in KRAS, a common protooncogene encoding for a small GTPase, are found in approximately 13% of colorectal cancers and in 45% of pancreatic cancers [72, 73]. The most common *KRAS* mutations are gain-of-function mutations known as “hot-spot” driver mutations [74], with the most frequent one being a substitution of the amino acid glycine with aspartic acid at codon 12, denoted as *KRAS* G12D [74]. Despite decades of investigation, researchers and clinicians still have not developed a drug or vaccine that can effectively target the KRAS protein in humans [74]. However, recent research has indicated that lymphocytes may be used as a viable source of T cells to combat tumorigenicity via T cell receptors specific to the patient and mutation types [75].

Tran et al. exemplified this by demonstrating that the transfer of T cells, with T cell receptors specific to KRAS G12D, can have profound antitumorigenic effects on specific cancer types [74]. In this occurrence, T cells containing the pertinent T cell receptor were so selective that they could distinguish the mutant, oncogenic KRAS G12D from the wild-type KRAS despite just a single point mutation. Their studies involved a 50-year-old patient who had metastatic colorectal cancer. Throughout the course of her T cell therapy, it was observed that CD8^+^ T cells were reacting with the HLA-C alleles associated with the mutant KRAS G12D peptide known as the HLA-C∗08:02 allele. This manifested itself as a marked regression in tumor size. Of the four KRAS G12D-reactive T cell receptors generated, all reacted with the mutant peptide and most importantly none of the receptor types reacted with wild-type KRAS.

The researchers reported that after 40 days post-T cell therapy, all seven metastatic tumors had regressed. Nine months posttherapy, 6 of the 7 tumors had completely regressed or showed continuing regression [74]. Thus, this T cell therapy represents a potential tumor cell-specific recognition technique, uniquely targeting tumor cells expressing the KRAS G12D mutation and the HLA-C∗08:02 allele. The conclusion of the study was that thousands of patients per year could potentially benefit, if qualified, from this T cell-based immunotherapy targeting KRAS G12D.

One limitation of TIL therapy is logistical, as the process of cultivating the cells is laborious and time consuming. Treatment with TIL therapy requires an average dose of 30–60 × 10^9^ cells, which are difficult to grow in a short timeframe [76]. Tumor resection to growing an appropriate quantity of infusible lymphocytes takes approximately 5-6 weeks [66]. The other drawback is its lack of versatility, namely, its limitation to predominantly just metastatic melanoma. To alleviate this issue, transduction of tumor-specific TCR genes into autologous T cells has been implemented to increase the repertoire of endogenous T cells.

### 3.2. TCR-Transduced T Cells

TCR-transduced T cells are often generated via genetic induction of tumor-specific TCR. This is often done by cloning the particular antigen-specific TCR into a retroviral backbone. Blood is drawn from patients and peripheral blood mononuclear cells (PBMCs) are extracted. PBMCs are stimulated with CD3 in the presence of IL-2 and then transduced with the retrovirus encoding the antigen-specific TCR. These transduced PBMCs are expanded further in vitro and infused back into patients (Figure 2(c)) [77].

TCR-transduced T cells present many advantages and solutions to other immunotherapies. Firstly, and most importantly, there is a robust ability for TCR-transduced T cells to be generated against a plethora of tumor antigens [78]. Secondly, engineering approaches (i.e., inclusion of disulfide bonds and TCR-optimized genes) allow for an easily enhanced expression of the modified TCR [79, 80]. Finally, TCR-transduced T cells are able to circumvent self-tolerance to defined self-antigens and persist for a long term in vivo [81]. Although seemingly versatile and robust, one major hurdle is that transferred TCR chain can unintentionally pair with endogenous TCR chains resulting in mispaired dimers and thus decreased reactivity [82]. In order to circumvent these problems, an experiment utilizing modern CRISPR-mediated gene editing was used to knockout endogenous TCR chains to increase surface expression of the modified TCR [83]. CD4 and CD8^+^ T cells were redirected more effectively in patient-derived B acute lymphoblastic leukemia compared to standard TCR transfer showing a stronger response and more sensitivity towards the tumor antigen [83].

One tumor antigen of particular interest is NY-ESO-1, a cancer germline antigen. This antigen is expressed in approximately 70–80% of synovial cell sarcoma and 25% of melanoma patients [77]. In a pilot trial, autologous peripheral blood mononuclear cells (PBMCs) were retrovirally transduced with NY-ESO-1-specific TCR and infused into patients bearing metastatic melanomas and metastatic synovial cell sarcomas. 11/18 patients with synovial cell sarcoma and 11/20 patients with melanoma demonstrated an objective clinical response [77].

### 3.3. Chimeric Antigen Receptor T Cells (CAR T Cells)

**Figure 3:**
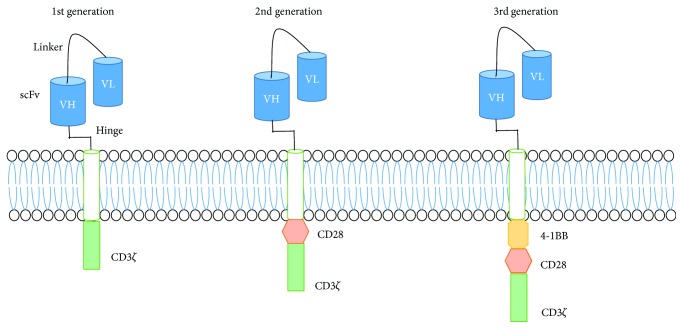
CAR T cell structure and design. Structure of first-, second-, and third-generation engineered CAR T cells. Each generation consists of the variable heavy and variable light portion of the antibody, with a hinge domain and CD3*ζ*. In each subsequent generation, an additional costimulatory molecule (i.e., CD28 or 4-1BB) is supplemented.

Adoptive transfer of chimeric antigen receptor (CAR) T cells, T cells expressing an engineered receptor designed to guide T cells towards tumor cells, has shown remarkable success for the treatment of acute and chronic B cell leukemias. CAR T cells, specifically, consist of an extracellular single-chain variable fragment (scFv) linked, via a hinge domain, to an intracellular CD3*ζ* which allows the T cell to recognize a cell surface tumor antigen with the affinity of an antibody coupled with the effector capability of T cells. CAR T cells have evolved though many generations to include multiple costimulatory signaling domains to enhance survival/proliferation (Figure 3). In addition to B cell leukemias, it has shown promise in improving recovery from allogeneic hematopoietic stem cell transplantation (alloHSCT) treatment.

Many patients with B lymphocyte malignancies have been treated with alloHSCT and successfully recovered from their malignancies [84]. During an alloHSCT treatment, a patient receives hematopoietic stem cells intravenously after chemotherapy or radiation therapy; the infused hematopoietic stem cells continue to differentiate into leukocytes and erythrocytes [85]. However, it has been discovered that many of the patients that were treated with alloHSCT either relapsed to their previous condition or did not enter complete remission. More specifically, patients with acute lymphoblastic leukemia (ALL) had a 5.5-month median survival rate, and patients with diffuse large-B cell lymphoma (DLBCL) had the disease persisting even after the alloHSCT treatment [84].

The malignancies that persisted or returned after an alloHSCT treatment are usually treated with DLIs. During these treatments, patients receive an infusion of lymphocytes from their alloHSCT donors. The infusions are given in the hope of mounting a graft-versus-tumor response in the patient. One third of the patients that receive DLIs develop acute graft-versus-host disease (aGVHD); this disease in turn causes mortality in 6–11% of patients treated with DLIs. DLIs are ineffective at treating patients with ALL and DLBCL, and an alternative treatment method should be employed after patients have been treated with alloHSCT [84].

Patients infused with allogeneic T cells that are expressing B cell-specific anti-CD19 CARs have been shown to have better recovery after an alloHSCT treatment [84]. T cells can thus be transduced to recognize CD19 receptor located on B cells and eliminate them from the body. The T cell receptors can be genetically modified to express chimeric antigen receptors (CARs) [86], which only recognize CD19 and thereby produce a graft-versus-malignancy response. In a study conducted by Brudno et al., 20 patients with B cell malignancies were treated with CAR19 T cell infusions. The results of the study demonstrated that none of the patients developed acute GVHD, although GVHD did occur in two of the patients. One of the patients developed mild chronic ocular GVHD two years after the infusion, at which point the CAR19 T cells would be no longer present in the body. The other patient had chronic GVHD, which progressively worsened [84].

The study determined that eight of the twenty patients obtained complete remission or partial remission. These patients had higher peak blood CAR19 T cell levels than did patients that had stable or progressive disease, and the peak CAR19 T cells were independent of the concentration of the CAR19 T cells that were infused in the patient [84]. Furthermore, the CD8 : CD4 ratio was higher in patients that had obtained partial or complete remission as compared to patients that did not [84]. The four subsets of T cells were also shown to be present at different concentrations during and after the CAR19 T cell infusions [84]. More naïve and central memory T cells were expressed in the infused CD19 cells; these T cells are less differentiated and have a larger capacity to proliferate [84]. After the infusion, these less differentiated cells were replaced by effector memory and effector RA T cells [84]. Naïve and central memory T cells lack inflammatory and cytotoxicity function [87], whereas the effector memory and effector T cells contain a plethora of chemokine receptors that allow them to infiltrate inflamed tissues [88]. The study also discovered an increase in CAR19 T cells expressing a programmed cell death protein-1 (PD-1) receptor during the peak CAR19 T cell levels [84]. Since patients that had achieved complete remission or partial remission had higher peak CAR19 T cell levels than did patients that had stable or progressive disease, elevating the CAR19 T cell levels in patients might lead to better treatment results. These findings can be used to improve future treatment options, such as creating vaccines that will aid in increasing the peak CAR19 T cells during treatment or developing PD-1 antagonists [84].

To elaborate on the success of CD19 CAR T therapy, an interim analysis of phase II testing of CD19 CAR T cell therapy drug, KTE-C19, in patients with DLBCL showed a high response rate. In the trial, 76% of 51 patients showed a response to treatment, 47% had a complete response, and after 3 months, 33% continued to experience a complete response [89]. CAR T cell therapy proved to be just as successful in clinical trials as it was on the benchtop, especially with the recent FDA approval of tisagenlecleucel to treat acute lymphoblastic leukemia (ALL).

CAR T-related toxicities, such as cytokine release syndrome, need to be addressed in order for CAR T cell therapy to become a widely used treatment option. Cytokine release syndrome (CRS) is a phenomenon described after administration of modified T cells by which a storm of inflammatory cytokines, primarily IL-6, IL-10, IFN-*γ*, is released [90]. Often times, this results in mild flulike symptoms; however, symptoms including hypotension, pulmonary edema, multiorgan failure, and CRS-related death have been reported [91]. In order to counteract and mitigate CRS, corticosteroid treatment and IL-6 blockade treatments have been implemented. Corticosteroids, in the context of CAR T cell-related CRS, have been controversial. After immediate corticosteroid administration, a dramatic decrease in elevated inflammatory cytokines is observed, but at the cost of a partial response to CAR T cell treatment [92]. A more viable solution is manipulating IL-6, which is implicated in a variety of immune-related processes (neutrophil trafficking, B cell differentiation, etc.). IL-6 blockade, using the FDA-approved tocilizumab, has resulted in rapid reversal of life-threatening CRS while maintaining the efficacy of CAR T cell treatment [93].

Another barrier for CAR T cells is their inability to penetrate and permeate through a solid tumor microenvironment. For this reason, CAR T cells are currently being evaluated and modified for successful use in solid tumors (such as pancreatic cancer). Research is underway to improve the potency of CAR therapy in solid tumors. One method involves the administration of drugs such as fludarabine to inhibit IDO, an enzyme that naturally degrades tryptophan in the tumor microenvironment, in turn regulating T cell activity [94]. Another route is through cytokine manipulation, particularly IL-12 inflammatory cytokine, which is able to induce a Th_1_ response and initiate CD8 T cell clonal expansion [95]. Research is underway to alleviate these limitations of CAR T cell therapy to allow the engineered T cell to move unperturbed throughout the dense solid tumor environment.

## 4. Bispecific Antibodies

Tumor-infiltrating lymphocytes and checkpoint inhibitors are aimed at using cell markers to target and kill cancer cells directly; however, alternative research methods highlight the potential of using biological therapeutics as another method for evading cancer metastasis [96–98]. Biologically directed cancer therapeutics can be used to prevent or diminish continued cancer cell proliferation by upregulating the activity of the host immune system through the use of engineered antibodies to target known molecules within oncogenic pathways rather than by acting on cancer cells directly [99, 100].

The first monovalent antibody (mAb) approved by the FDA was rituximab (Rituxan), an anti-CD20 antibody (mAb) for the treatment of hematologic malignancies [99]. Despite the success of rituximab, most mAbs stimulate redundant signaling pathways that can promote cancer cell survival, among other limitations [99]. However, the inadequacies of monovalent antibodies have been overcome by the advent of bispecific antibodies (bsAbs) with dual antigenic specificities, which are capable of simultaneously interacting with multiple receptors and/or ligands [99]. Currently, there are ongoing efforts to design novel bsAbs that can target various forms of human malignancies to enhance the therapeutic potential of antibody treatment.

### 4.1. Bispecific Antibodies to Engage T Cells

The large issue with monoclonal antibodies, which highlights the benefits of bispecific antibodies, is the suboptimal interaction with effector cells due to alternative Fc glycosylation or Fc receptor polymorphisms [99]. The benefits of bispecific antibodies (bsAbs) rely on their ability to target 2 unique cell types and direct immune effectors towards cancer cells. This has proven to be efficacious since bispecific T cell engagers (BiTE), bispecific antibodies that recruit T cell effectors, have become a valid therapeutic in the treatment of a number of cancers. One BiTE, blinatumomab, is a CD3/CD19 bispecific antibody that has recently been approved by the FDA for the treatment of acute lymphoblastic leukemia by targeting the TCR on T cells (CD3) and recruiting them to B cells (CD19) [101]. On the other hand, the monoclonal antibody (mAb) rituximab, an anti-CD20 mAb, is the current gold standard in B cell lymphoma treatment. Anti-CD20 is the preferred B cell marker, compared to CD19, for many reasons including (1) antibody-dependent cell-mediated cytotoxicity, (2) complement-dependent cytotoxicity, (3) antibody-mediated phagocytosis, and (4) cancer cell apoptotic ability. However, issues exist with the standalone anti-CD20 mAb, mainly cancer relapse and tumor metastasis after treatment, due to incomplete depletion of B cell and drug resistance [102]. To ameliorate these issues, a tetravalent anti-CD20/CD3 bispecific antibody was developed and examined preclinically for the treatment of B cell lymphoma [103].

The research team produced a fully functional bispecific antibody consisting of an anti-CD20 molecule, 2 single-chain variable fragments (scFv) of anti-CD3, and a linker hinge domain (LHD). The anti-CD20 is specific for B cells, while the anti-CD3 scFvs are specific for T cells. The LHD improves stability to the CD20 C-terminus and stabilizes the scFv fragments by forming disulfide bonds between IgG1 heavy chains. After production and size exclusion chromatography, a 97% purity indicated a lack of aggregation and thus potential to improve production yields and solubility. Beyond that, a cytotoxicity assay was performed comparing this new bispecific antibody to rituximab. It was determined that this new antibody had a 6 times higher cytotoxicity potential than the current standard rituximab. With improved stability, solubility, production yield, and cytotoxicity, this tetravalent anti-CD20/CD3 bispecific antibody may provide an alternative to patients with CD20^+^ and rituximab-resistant B cell malignancies. Preliminary studies involving this anti-CD20/CD3 BiTE merely touched the surface with in vitro studies. To accurately and thoroughly determine efficacy and potential, future studies of pharmacodynamics/kinetics and systemic effects must be done.

**Figure 4:**
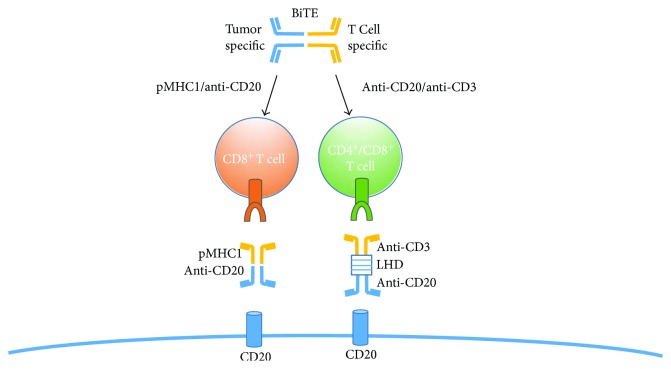
A general schematic outlining the structure of bivalent T cell engager (BiTE) antibodies. One end of the bispecific antibody is against a B cell tumor antigen (CD20), and the other end is specific against T cells (pMHC1 or CD3). The bivalent antibodies help direct T cells toward the vicinity of the tumor to aid in cytotoxic destruction of the neoplasm.

Other more specific methods involving BiTE molecule technology have been developed, involving the cytomegalovirus (CMV), a prevalent herpes virus found in the vast majority of the population. CMV, to people with normal immune function, is nonpathogenic as invasive T cell populations (CD8^+^ and CD4^+^ cells) infiltrate and maintain homeostasis [104]. Postinfection, these CD8^+^ T cells become memory-like and get sequestered in peripheral tissue yet remain functional [104]. These antigen-experienced CD8^+^ T cells can go on to recognize MHC class I—peptide complexes on the surface of target cells [105]. By coupling an antitumor antibody fragment to an MHC molecule loaded with a viral peptide (i.e., CMV peptide) and coating the tumor with the complex, a tumor can resemble a virally infected cell, thus marking it for destruction by CVM-specific CD8^+^ T cells (Figure 4) [106–108]. Tumor cells have evolved to evade the immune system by mechanisms such as loss of antigenicity and suppression of local effector T cells. The BiTE-like molecule (pMHC1-viral/antitumor antibody) described here can aid in overcoming this evasion and selectively engage antigen-specific T cells as opposed to other BiTEs which ubiquitously activate T cells [107–109].

It has now been shown that in order to create a successful complex, one must add the pMHC1 complex to only one of two antibody chains, in particular, the unfused heavy chain with a “knob” mutation in order to produce less side products [105]. This technology allows for the expression of heterodimeric IgGs with two dissimilar heavy chains [105]. MHC1 complexes are said to be not as stable as antibodies [110], and by inserting an artificial disulfide bond in the space separating the linker of the MHC peptide and the MLA heavy chain [111, 112], the first melting point can be increased and less aggregates can be produced [105]. This allows for more product to be generated while mitigating any excess, unwanted products. This proposed complex offers the opportunity for monovalent antibody binding [105] and the delivery of practical peptide-MHC class I complexes to tumor cells. The proposed pMHC1-fused IgG antibodies can initiate CD8^+^ T cells to squander tumor cells at sub-nanomolar amounts and at a smaller effector to target cell ratio [105]. pMHC1-IgGs can accompany cancer immunotherapy in redirecting internal antigen-specific T cells allowing for improved treatment outcomes.

### 4.2. Bispecific Antibodies to Target MET-Expressing Cancers

Bivalent antibodies have also been designed to target MET-expressing cancers. MET (mesenchymal-epithelial-transition factor) is a receptor tyrosine kinase (RTK). RTKs regulate a diverse array of cellular processes involved in tissue homeostasis [113]; however, the dysregulation of RTKs has been linked to the development and progression of various human carcinomas [114, 115]. Therefore, not only are RTKs essential mediators of normal physiology but these receptors also serve as attractive therapeutic targets for selected cancers [114].

MET is expressed predominately on epithelial cells and is a high-affinity receptor for only one known ligand, hepatocyte growth factor (HGF) or scatter factor [115, 116]. HGF is secreted by mesenchymal cells and induces c-MET dimerization and subsequent activation of MAPK, PI3K/AKT, STAT3/5, and Ras/Raf downstream signaling cascades [114, 117–119].

Consequently, the aberrant activation of this HGF/c-MET signaling pathway promotes tumor cell proliferation and angiogenesis, inhibition of apoptosis, and EMT programming and is linked to an overall poor patient prognosis and survival rate [113, 120, 121]. In addition, an overexpression of c-MET protooncogene has been implicated in the development of acquired resistance to RTK inhibitors [118, 122, 123]. This fact is alarming given that HGF/c-MET expression occurs in most carcinomas and is associated with the invasive phenotype of several forms of tumors including breast, lung, renal, gastric, and hepatocellular carcinoma [113, 115, 124, 125].

Furthermore, HGF/c-MET signaling can be initiated by both ligand-dependent and ligand-independent mechanisms [113, 116]. HGF binding mediates ligand-dependent activation, whereas the amplification of the c-MET oncogene mediates ligand-independent activation [113, 116], which results in c-MET overexpression and subsequent receptor dimerization and cross-activation [113, 124, 125]. Like most RTKs, c-MET demonstrates a propensity to participate in cross-talk (cross-activation) with other families of receptors, such as epidermal growth factor receptor (EGFR) [122, 126]. Consequently, tumors that express both c-MET and EGF receptors invariably develop acquired resistance to EGFR tyrosine kinase inhibitors (EGFR TKIs) due to ligand-independent c-MET activation [121]. Therefore, dysregulation of the HGF/c-MET pathway can occur if either HGF or c-MET expression becomes elevated due to gene rearrangements or mutations [113]. Given that the HGF/c-MET signaling is involved in several processes underlying tumorigenesis, inhibition of this pathway is an obvious therapeutic approach against c-MET-expressing cancers such as NSCLC [121]. There are several strategies through which c-MET activation can be inhibited, including interference with HGF binding, c-MET dimerization, c-MET kinase activity, and downstream signaling [122, 125].

Currently, immunotherapeutic approaches have emerged as the preferred method to c-MET inhibition due to the low toxicity and increased specificity of antibody therapies relative to classical cytotoxic agents and new molecular target agents (MTAs) [127, 128]. Although numerous monovalent anti-HGF and anti-MET antibodies have proven to be efficacious in clinical trials, these antibodies can only inhibit ligand-dependent c-MET activation [129] and hence are insufficient at preventing EGFR TKI resistance that occurs due to ligand-independent c-MET activation. This shortcoming of monovalent anti-HGF and anti-MET therapeutics can be overcome through the application of bispecific antibodies which not only combine the specificity of two antibodies but also permit the simultaneous targeting of different antigens or epitopes resulting in more precise targeting [97, 99, 117, 130, 131].

**Figure 5:**
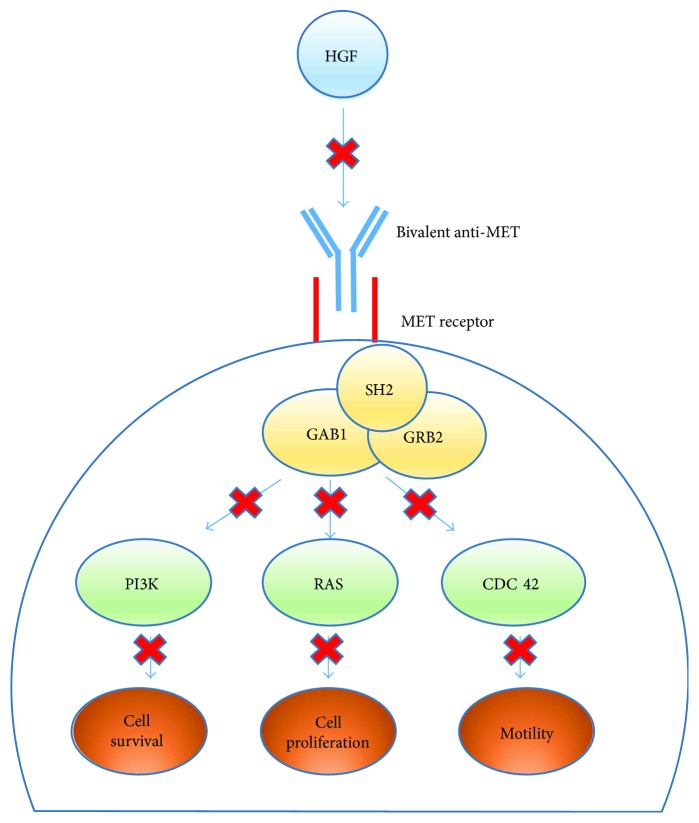
Bivalent design and function of anti-MET antibodies and siRNA aptamers. A bivalent anti-MET antibody blocks the HGF ligand from binding to the dimerized MET receptor. This prevents the activation of a multitude of factors required for tumor cell survival, proliferation, and motility.

Emibetuzumab is a humanized, bivalent anti-MET antibody designed to inhibit both ligand-dependent and ligand-independent activation of the HGF/c-MET pathway via a dual mechanism of action [120, 129]. Emibetuzumab inhibits ligand-dependent activation by binding to the extracellular domain of c-MET and blocking HGF binding thereby preventing HGF-induced c-MET phosphorylation and cell proliferation (Figure 5) [129, 132]. In addition, emibetuzumab induces c-MET internalization and degradation thus inhibiting constitutive c-MET activation due to receptor overexpression [129]. Early generations of bivalent anti-MET antibodies have been largely unsuccessful because these antibodies exerted an agonistic effect causing proliferation of both normal cells and tumor cells [129, 132]. However, emibetuzumab is unlike other anti-MET antibodies because it does not demonstrate functional agonistic activity and therefore cannot elicit an HGF-related response [120]. Yoh et al. and Lui et al. demonstrated that emibetuzumab has potent antitumor activity and is capable of successfully targeting MET-expressing tumors driven by HGF overexpression and constitutive c-MET activation without exerting an agonistic effect [120, 129].

These findings were further substantiated by a phase I clinical trial in which emibetuzumab was administered both as a monotherapy and in combination with EGFR TKIs, erlotinib, and gefitinib [120]. In the combination therapy cohort, 4 out of 6 patients with NSCLC, who had been previously treated with first-generation EGFR TKIs and were shown to have developed resistance, experienced a best response of SD and experienced a PFS range of 50 to 174 days. Patients who experienced the longest PFS range and/or maintenance in tumor baseline correlated with an increase in the number of emibetuzumab treatment cycles [120]. Furthermore, no dose limiting toxicities (DLTs) or severe adverse effects (SAE) were observed in relation to emibetuzumab administration, thus confirming the safety and tolerability profile of this c-MET inhibitor both as a monotherapy and in combination with EGFR TKIs [120].

The validity of bivalent anti-MET antibodies as therapeutic agents against c-MET is demonstrated by the successes of emibetuzumab clinical trials. Bivalent antibodies have shown promising potential as biological therapeutics capable of inhibiting tumor growth driven by elevated HGF as well as by constitutive activation of c-MET through overexpression, gene amplification, or genetic mutation.

## 5. Conclusion

The advancements made in the field of immunology have stimulated development of many new therapeutics that have tremendous potential for treating human cancers. Though future research needs to be conducted in order to further understand the mechanisms of drug-induced toxicities, these new therapies have resulted in improved cancer treatments (regression, remission, and overall survival).
